# Biomechanical Influence of Different Cervical Micro-Thread Forms over Narrow-Diameter Implants (2.9 mm) Using Finite Element Analysis

**DOI:** 10.3390/jfb16110420

**Published:** 2025-11-11

**Authors:** Qiannian Zhang, Waikit Lau, Nalini Cheong, Tonghan Zhang

**Affiliations:** 1 Hospital of Stomatology, Zhongshan City, Zhongshan 528400, China; hfdplt@stu2023.jnu.edu.cn (Q.Z.); lwjkq@stu2022.jnu.edu.cn (W.L.); saphira112811@gmail.com (N.C.); 2 School of Stomatology, Jinan University, Guangzhou 510632, China

**Keywords:** finite element analysis, small diameter dental implants, face angle, thread form, microstrain-stress distribution

## Abstract

Narrow-diameter implants (≤3.5 mm) have garnered significant attention due to their widespread application in areas with insufficient bone volume. However, their mechanical performance is limited. The cervical region, serving as a pivotal stress concentration zone, exhibits a thread form that directly modulates stress distribution and determines the long-term stability of the implant–bone interface. This study was designed to investigate the influence of varying thread forms and face angles on microstrain and stress distribution patterns in narrow-diameter implants (NDIs) and their adjacent cortical bone structures. Through systematic modification of implant thread forms and face angle parameters, finite element analysis (FEA) was employed to develop nine distinct implant models featuring varied geometric characteristics. Each model was implanted into Type III bone tissue, followed by the application of a 100 N occlusal force, including a vertical load and an oblique load deviated 30 degrees lingually from the long axis of the implants. Subsequent biomechanical evaluation quantified peak von Mises stress concentrations at the bone–implant interface, maximum equivalent elastic strain distributions in peri-implant bone tissue, and abutment stress profile characteristics. The results indicated that in the RB thread group, the optimal thread face angle parameter was 60 degrees; in the B thread group, this optimal thread face angle parameter was 45 degrees, whereas in the V thread group, the optimal thread face angle parameter was 30 degrees.

## 1. Introduction

With the widespread adoption of oral implantology, clinicians often face the challenge of insufficient alveolar bone width. The emergence of narrow-diameter implants (≤3.5 mm) not only provides a minimally invasive treatment option for elderly patients with poor physical conditions, helping them avoid complex bone augmentation procedures, but also reduces the financial burden on patients and lowers the incidence of intraoperative and postoperative complications [1,2]. However, the reduction in diameter also raises biomechanical concerns. According to engineering mechanics principles, a decrease in cross-sectional area leads to a substantial elevation of internal stress when the implant is exposed to an equivalent occlusal load. It also increases the load on the surrounding bone tissue, which may result in fatigue fracture of the implant or bone resorption due to excessive loading [3,4].

**Figure 1:**
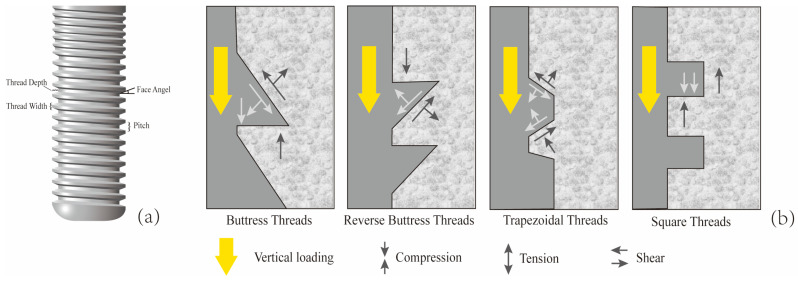
Characteristics of implant thread parameters. (**a**) Face angle: the angle between the threaded surface and the perpendicular line to the long axis of the implant. Pitch: The distance from the center of one thread to the center of the next along the longitudinal axis of the implant, divided by the number of threads. Thread depth: The distance between the outer contour of the thread and the base of the implant. Thread width: The distance between the crest (top) and the crest (top) of the same thread; (**b**) When the implant is subjected to vertical loading, the stress conditions at the implant–bone interface for four different thread forms (Mod according to [13,16]).

The implant neck is the transitional area connecting the abutment to the main body of the implant, and it is also a critical hub for stress transmission. Both in vitro experiments and biomechanical analyses have shown that this region is the most prone to stress concentration [5,6]. Excessively elevated stress at the implant neck constitutes a primary risk factor contributing to marginal bone loss (MBL) in the peri-implant region. Moreover, once MBL occurs, it further increases the stress borne by the implant neck [7,8]. This not only affects the final aesthetic outcome of the implant but may also trigger peri-implantitis, ultimately resulting in implant failure [9,10]. A previous FEA study showed that peri-implantitis is often accompanied by cortical bone resorption and even cortical bone loss. The absence of cortical bone can lead to higher stress loads on the bone tissue [11]. As a critical determinant of cervical implant biomechanics, thread design governs peri-implant stress distribution patterns and directly modulates primary stability [12]. Each thread unit comprises multiple geometric parameters, such as thread form, depth, width, pitch, and face angle (as shown in Figure 1a) [13]. The thread forms include trapezoidal thread (V thread), square thread (S thread), buttress thread (B thread), and reverse buttress thread (RB thread) (Figure 1b). Although there is already a substantial body of literature employing FEA to study the effects of different thread forms on the stress distribution in implants and cortical bone, the conclusions drawn from these studies have not yet reached a fully unified consensus. For example, Liu et al. demonstrated through FEA that among the four compared thread forms, the buttress thread can achieve a more uniform stress distribution in the cortical bone [14], whereas Mansi et al. found that thread form had no correlation with cortical bone stress and that implants with reverse buttress threads produced the least stress in cancellous bone compared to other thread types [15]. The face angle is commonly defined as the angle between the threaded surface and the perpendicular line to the long axis of the implant (Figure 1a). Critically, uniform thread configurations may still yield markedly distinct biomechanical responses in peri-implant bone when face angle parameters differ [16].

The cervical micro-threading system incorporates micro-scale thread profiles at the cortical bone interface, exhibiting constrained thread pitch and deliberately limited depth of thread engagement. Existing clinical evidence suggests that implants with micro-threading may outperform those without such designs in maintaining marginal bone levels [17]. Biomechanical analyses also theoretically support the idea that micro-threading can increase the surface area at the neck, thereby reducing peak interfacial stress [18]. However, current research on implant thread form and face angle primarily focuses on standard-diameter implants. There is not only a lack of direct comparative studies on the thread form of various NDIs, but also few studies that conduct a joint analysis of thread form and face angle parameters. Notably, the micro-threads at the neck of NDIs are in direct contact with cortical bone, making them more prone to stress concentration effects and significantly increasing the risk of neck fracture. Nevertheless, the current literature has yet to reach a clear consensus on which combination of thread form and face angle parameters for the neck micro-threads can achieve optimal biomechanical performance between the implant and the surrounding bone tissue.

FEA, as a mature and powerful virtual experimental technique, enables researchers to systematically and non-invasively evaluate the effects of minor adjustments in implant design parameters, different occlusal loading conditions, and individual anatomical variations among patients on the biomechanical response of the entire implant system within a computerized environment. For example, through finite element modeling, Li and colleagues quantitatively assessed the relationship between implant insertion depth and resultant bone stress profiles in the context of short implant applications [19]. Similarly, Yağır and collaborators utilized FEA to comparatively assess how variations in abutment material composition and geometric configurations influence mechanical stress profiles in peri-implant bone structures [20]. Therefore, this study would employ the FEA method to construct implant models with different neck micro-thread forms and face angles. It would systematically compare the biomechanical performance of implants with various neck designs—RB thread, B thread, V thread, and S thread—under simulated physiological functional loads. The research aimed to elucidate: which neck thread form is most conducive to reducing stress concentration in the marginal cortical bone of narrow-diameter implants? And how do different thread forms and face angle parameters influence the transmission and distribution of stress along the implant–bone interface? The null hypothesis of this study was that there are no differences in the effects of varying micro-thread forms and face angle parameters on the microstrain-stress distribution of narrow-diameter implants (2.9 mm) and their surrounding bone tissue.

## 2. Materials and Methods

**Figure 2:**
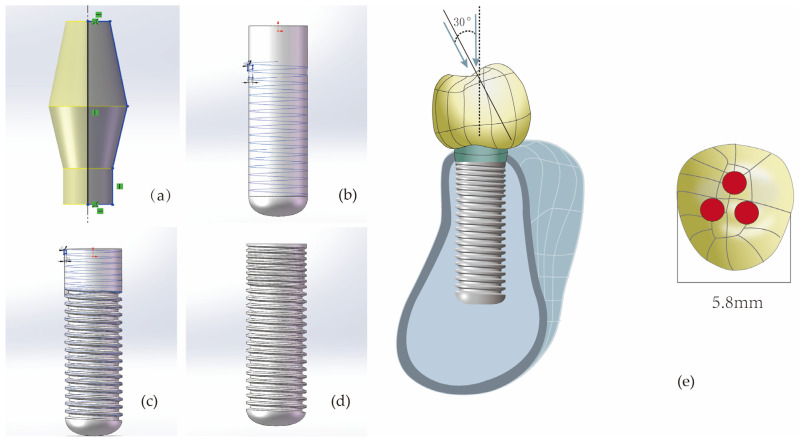
The modeling process of implant components (taking V-shaped threads with a face angle of 30 degrees as an example). (**a**) Constructing the abutment with set parameters; (**b**) Cutting the non-neck portion of the implant with an isosceles triangle having a base angle of 70 degrees to achieve V-shaped threads with a face angle of 20 degrees. (**c**) Cutting the neck portion of the implant with an isosceles triangle having a base angle of 60 degrees to achieve V-shaped threads with a face angle of 30 degrees. (**d**) The final implant model. (**e**) For every model, whether under vertical or inclined loading, the loaded area is the red region. In the picture, the area of one red circular region is 0.8 mm^2^.

By altering two design parameters of the implant (thread form and face angle), a total of nine implant models were obtained: M1 (RB thread, face angle 30 degrees), M2 (RB thread, face angles 45 degrees), M3 (RB thread, face angle 60 degrees), M4 (B thread, face angle 30 degrees), M5 (B thread, face angle 45 degrees), M6 (B thread, face angle 60 degrees), M7 (V thread, face angle 30 degrees), M8 (V thread, face angle 45 degrees), M9 (S thread, face angle 90 degrees). The total length of all implants was 10 mm, with a fixed diameter of 2.9 mm. The length of the micro-threaded neck portion was 2.2 mm, and the pitch was 0.3 mm. Due to the diameter limitations of narrow-diameter implants and the wall thickness constraints in the neck region, the thread depth at the neck was set to 0.1 mm. The remaining thread parameters for the non-neck regions remained consistent, specifically including: a pitch of 0.4 mm, a thread depth of 0.2 mm, a V thread structure, and a face angle of 20 degrees. The finite element models of the implants were constructed according to the above parameters using the solid modeling software program (SolidWorks 2021), and the specific process is illustrated in Figure 2. (During the actual model construction, due to limitations related to thread depth and pitch, the model with a V thread and face angle of 60 degrees was excluded.).

Next, this study utilized the 3D solid modeling software program (SolidWorks 2021) to construct a mandibular premolar bone block model (with cortical bone thickness of 0.75 mm, cancellous bone structure in the center, and material properties set to Type III bone density) [21,22], and an abutment (with an abutment taper angle of 12.5 degrees and a diameter of 3.75 mm). Subsequently, a reverse engineering software program (Geomagic Wrap 2021) was used to create a premolar crown model (with mesiodistal diameter of 5.8 mm), which was then imported into SolidWorks 2021 in STEP format to complete the part modeling. The implant component models were then positioned within the mandibular bone model in SolidWorks 2021 as an assembly. After eliminating interferences between the models through Boolean operations, the complete model was finally imported into the finite element analysis software program (ANSYS Workbench 2021 R1, ANSYS, Inc., Canonsburg, PA, USA) for biomechanical simulation.

In this study, all materials were assumed to be linear, elastic, homogeneous, and isotropic. Table 1 summarizes the material properties of the model components [23,24,25,26,27]. About the contact conditions, it was assumed that the interfaces between cortical bone and cancellous bone, as well as between bone and implants, were perfectly bonded to simulate ideal osseointegration; all other contact surfaces were also set to be bonded. After tetrahedral meshing, the total number of elements and nodes for models M1 to M9 are summarized in Table 2. To simulate clinically common functional loads, the study replicated two loading conditions: one involved applying a vertical load of 100 N (along the long axis of the implant) on the crown, and the other involved applying an oblique load of 100 N (angled 30 degrees toward the lingual side relative to the long axis of the implants) [19,28]. The specific loading site on the top of the crown is shown in Figure 2e. Finally, both sides of the mandibular bone block were fully fixed (restricting displacement in the x/y/z directions), with horizontal movement restricted on all four sides (only allowing minimal vertical micromovement).

**Figure 3:**
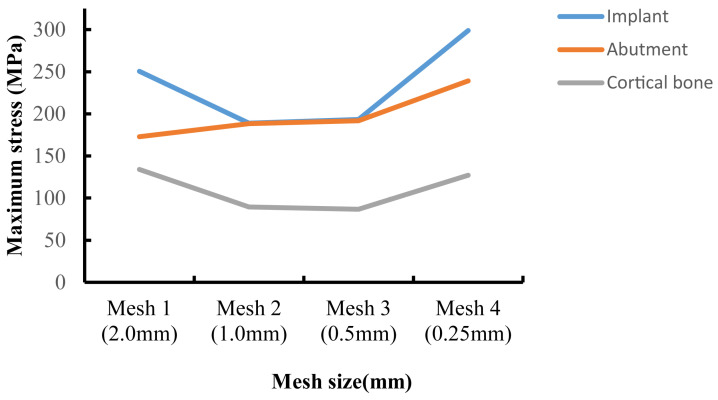
Convergence test results for maximum von Mises stress values.

Validating the finite element model constitutes a pivotal phase in FEA, with model accuracy serving as a critical parameter for assessing its predictive capability. Prior research has demonstrated that within the finite element domain, achieving comparatively reliable experimental outcomes is generally attainable when the model’s element and node count ranges between 30,000 and 200,000 [25,29]. However, this standard has significant limitations—for example, when using a larger mesh size for large-scale models, it may still result in a high number of elements and nodes. In contrast, employing mesh convergence analysis is a more reliable and objective validation method: by progressively refining the mesh until the stress variation rate of the implant-surrounding bone tissue falls below 5%, while simultaneously monitoring the color gradient changes in adjacent elements (with a focus on stress concentration areas). If the color difference between adjacent elements (especially in stress concentration zones) exceeds one color level, it indicates that the current meshing scheme is unreasonable [30,31]. In this study, the initial mesh size of each finite element component was set to 2 mm. The initial mesh size was progressively halved, and local mesh refinement was applied to stress concentration areas to enhance mesh quality. Figure 3 presents the mesh convergence curve for M8, indicating that when the mesh sizes of the abutment, implant, and cortical bone were refined to 0.25 mm, the error in the von Mises stress results exceeded 5%. Table 1 provides a detailed list of the final mesh size parameters for each component in this study.

This study implemented a rigorous computational framework to quantify how thread form and face angle parameters modulate the risk of implant-associated marginal bone resorption. High-fidelity biomechanical metrics—including maximum equivalent elastic strain and Von Mises stress—were analyzed to establish design-performance correlations. The abutment’s stress distribution was simultaneously modeled to assess the impact of thread geometry and face angle variations on its mechanical behavior.

## 3. Results

**Figure 4:**
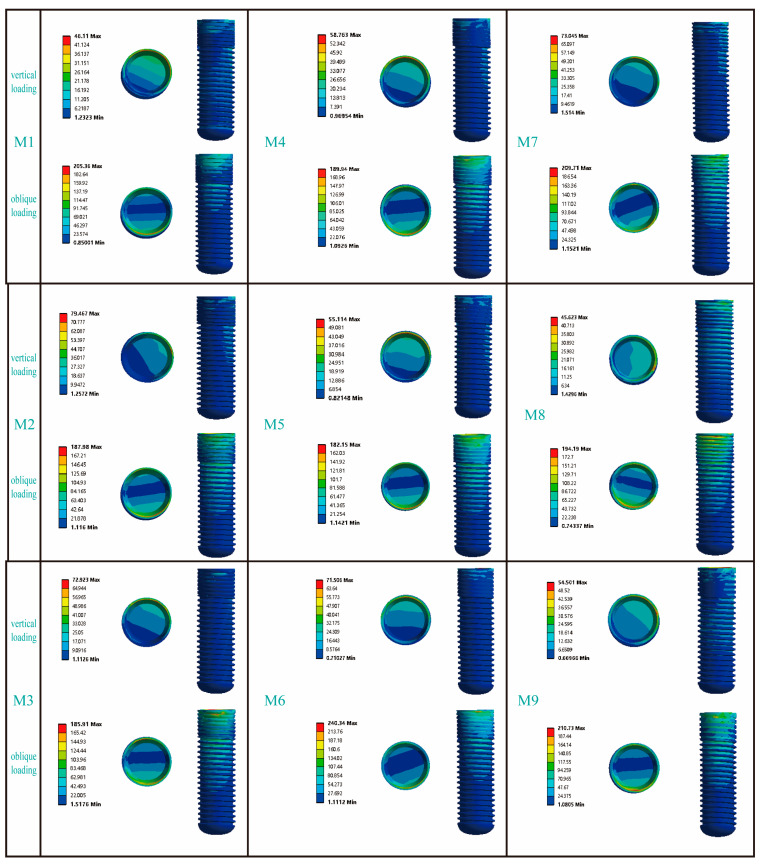
The influence of different thread form and face angle parameters on the stress distribution around the implant. In the figure, the circular patterns on the right side of the bars represent the stress distribution at the implant–abutment interface. The area indicated below refers to the buccal side of the implant.

Figure 4 presents the von Mises stress distribution of the implant within Type III bone. Under vertical loading conditions, the stress was predominantly localized at the implant-abutment interface, with notable concentrations in the mesial and lingual regions. While axial loading distributed stresses uniformly along the implant body, oblique loading induced focal stress concentrations at both the abutment coupling site and the coronal-most thread profiles—a phenomenon extensively validated in the literature [28,32]. Among all the models, the maximum Von Mises stress caused by oblique forces was greater than that caused by vertical forces. When M6 was subjected to an oblique force, the resulting von Mises stress was 240.34 MPa, which was the highest von Mises stress experienced by the implant in this study. In contrast, when M1 was subjected to a vertical load, the resulting Von Mises stress was 46.11 MPa, which was the lowest Von Mises stress experienced by the implant in this study. Comparisons between models showed distinct stress patterns. In the RB thread group (M1, M2, M3), M3’s stress under vertical load (72.92 MPa) was similar to M2’s but 36.8% greater than M1’s; under oblique load, its stress (185.91 MPa) was again comparable to M2 but 9.5% lower than M1. For the B thread group (M4, M5, M6), M6 carried the highest stress under both vertical (71.51 MPa, 17.8–22.9% higher than M4/M5) and oblique loading (240.34 MPa, 20.1–24.2% higher). Additionally, while M7 and M8 exhibited similar stress under oblique force, M7’s stress under vertical load (73.05 MPa) was 37.5% higher than that of M8.

**Figure 5:**
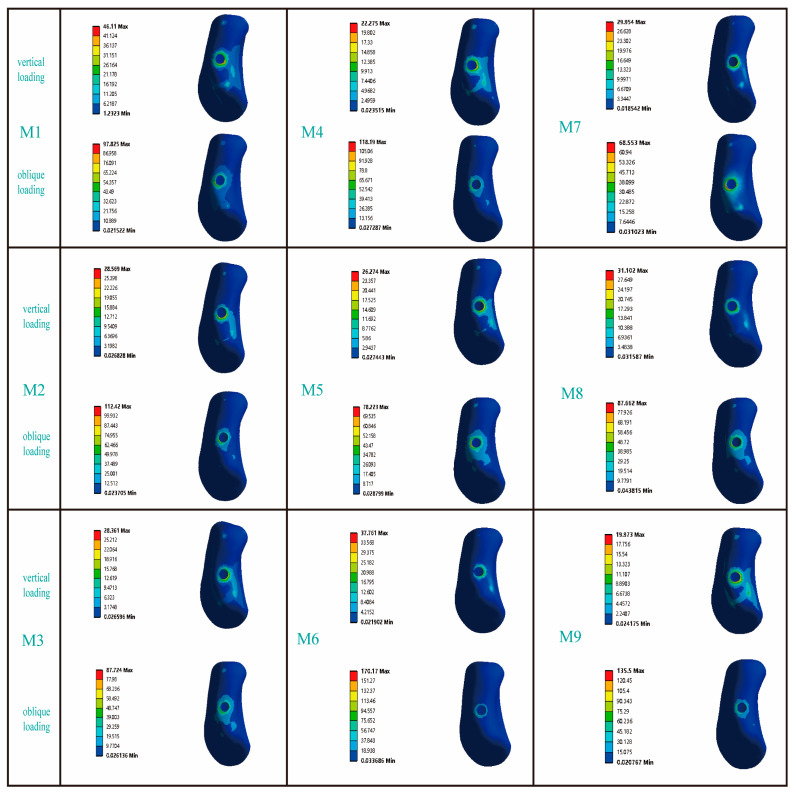
The influence of different thread form and face angle parameters on the stress distribution in cortical bone. The left side of the picture represents the buccal side of the jawbone.

Figure 5 presents the stress distribution patterns in the cortical bone adjacent to the dental implants. In response to vertical loading, cortical bone stress was predominantly concentrated in the mesial and lingual regions; conversely, when subjected to oblique loading, the primary stress localization occurred on the buccal side. Among the RB thread groups, the maximum von Mises stress in M3 under vertical loading was comparable to that of M2 but 14.9% higher than that of M1. Under oblique loading, however, the trend reversed: M1 and M2 exhibited 10.3% and 22.0% higher stress than M3, respectively. For the B thread groups, M6 consistently experienced the highest stress under both loading conditions. Vertically, its stress value of 37.76 MPa was 41% and 30.4% greater than that of M4 and M5, respectively; under oblique loading, its stress peaked at 170.17 MPa, exceeding M4 and M5 by 30.5% and 54.0%. Finally, while models M7 and M8 showed similar stress levels under vertical loading, M8’s stress under oblique loading (87.66 MPa) was 21.8% higher than that of M7.

**Figure 6:**
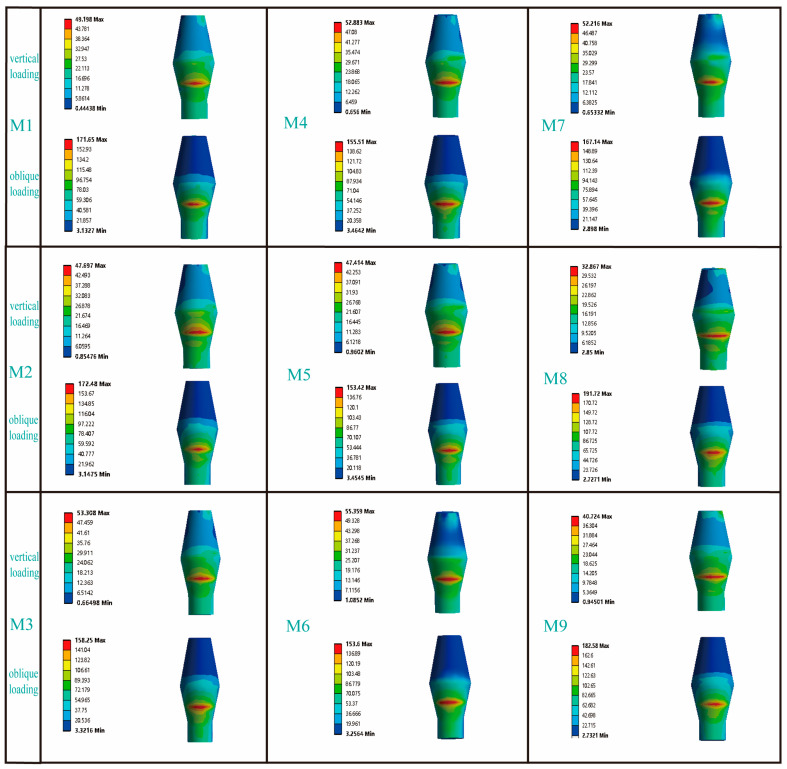
The influence of different thread form and face angle parameters on the stress distribution of the abutment. In the column for vertical loading, the picture shows the lingual side of the abutment, while in the column for oblique loading, the picture displays the buccal side of the abutment.

Figure 6 illustrates the distribution characteristics of von Mises stresses on the abutment. Under vertical loads, the stress was primarily concentrated in the area where the abutment contacted the lingual side of the implant; however, under inclined loads, the stress was mainly focused on the buccal side of the abutment. For the RB thread group, the maximum von Mises stresses on the abutments under both vertical and inclined loading conditions exhibited similar distribution patterns across the three models. Similarly, for the B thread group, the maximum von Mises stresses on the abutments under vertical and inclined loading conditions also showed similar trends. It was noteworthy that, in the V thread group (M7, M8), the maximum von Mises stress on the M7 abutment under vertical loading increased significantly by 37.1% compared to the M8 abutment; whereas under inclined loading conditions, the maximum von Mises stress on the M7 abutment decreased by 12.8% compared to the M8 abutment.

The equivalent elastic strain value of the jawbone is an important observation indicator in finite element experiments. Previous studies have shown that when the maximum elastic strain value of the jawbone exceeds 6.7 × 10^3^ με, an imbalance in the bone remodeling process around the implant will occur [33]. Table 3 presents the strain distributions in the cortical bone for all models. Under vertical loading, the strain values of all models were less than 6.7 × 10^3^ με, while under oblique loading, only M7 exhibited a maximum equivalent elastic strain value of 5.05 × 10^3^ ε (which is less than 6.7 × 10^3^ με). Table 4 summarizes the strain values of all implants under both vertical and oblique loading. According to previous studies, when the minimum and maximum equivalent elastic strain values of an implant fall within the range of 0.0015 to 0.0030 (mm/mm), it provides a favorable environment for bone growth [34]. Our findings indicated that in this study, the maximum equivalent elastic strain values of the implants across all models remained below 0.0030 (mm/mm).

## 4. Discussion

The different thread forms and face angle parameters of implants can influence the microstrain-stress distribution in the implants and surrounding bone, thereby refuting the null hypothesis. This study utilized finite element technology to evaluate the effects of different thread forms and face angle parameters on narrow-diameter implants. However, ensuring the reliability of the results from finite element studies remains an important consideration. Previous research has found that combining FEA with biomechanical models to simulate bone remodeling can assess the implant–bone interface closer to the real physiological environment [31]. Scholars such as S. Park combined FEA with biomechanical models to simulate the bone remodeling process and conducted a comparative study between the results and those from FEA that did not incorporate the bone remodeling mechanism. The findings indicated that for Type III bone, the results of FEA without considering the bone remodeling process can serve as a relatively conservative evaluation criterion; however, in Type IV bone, ignoring the bone remodeling process may lead to an underestimation of the actual risk level [35]. In this study, all materials were assumed to be isotropic. Therefore, the material properties were defined as Type III bone by adjusting the elastic modulus and Poisson’s ratio of the jawbone. Isotropic models remain widely used in previous and some recent studies [36,37]. However, extensive anatomical and biomechanical research has confirmed that bone tissue, particularly cortical bone, exhibits a distinct directional arrangement at the microscopic level (e.g., the Haversian system), leading to significantly anisotropic mechanical properties [38]. Consequently, establishing an anisotropic jawbone model can enhance the reliability of finite element analysis results. Nevertheless, the experimental determination of parameters for anisotropic jawbone models is highly complex. As a result, a simplified form—the orthotropic model—is commonly employed in practical research, as it is considered to more accurately represent the mechanical behavior of bone tissue [39]. Therefore, future research should further explore and apply the orthotropic model to enhance the credibility of FEA results and their clinical translational value.

**Figure 7:**
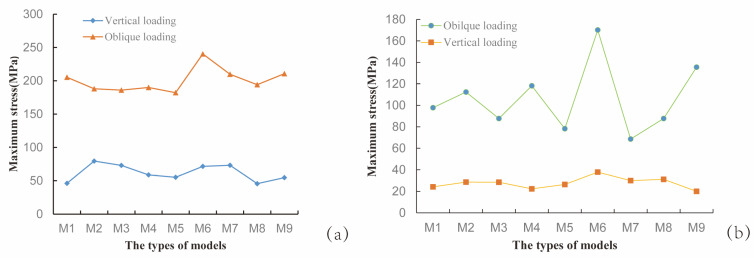
Maximum von Mises stress values for each model. (**a**) Stress in the implant; (**b**) Stress in the cortical bone surrounding the implant.

To investigate the influence of thread form and face angle parameters on the biomechanical properties at the implant–bone interface, this study focused on analyzing the von Mises stress distribution and equivalent elastic strain characteristics between the implant and cortical bone. As shown in Figure 7a, under inclined loading conditions, the maximum von Mises stress values for the RB thread implants exhibited a decreasing trend with increasing face angle; in contrast, under vertical loading conditions, the maximum von Mises stress values showed an increasing trend with increasing face angle. In both B and V thread groups, a progressive increase in the implant’s maximum von Mises stress was observed with rising face angle, though statistically significant only within a specific range. Similarly, under oblique loading, the M7 and M8 implants in the V thread group demonstrated similar stress values. As shown in Figure 7b, under oblique loading, the maximum von Mises stress in cortical bone initially increased and then decreased with increasing implant face angle in the RB thread group, while the opposite trend—first decreasing then increasing—was observed in the B thread group. In contrast, the V thread group showed a consistently rising stress trend with increasing face angle. This suggested that variations in thread form and their corresponding face angles exert distinct influences on the stress distribution within the cortical bone. Each thread configuration had an optimal face angle parameter that matched it, and the specific configuration of the thread form and face angle notably influenced the stress distribution patterns within both the dental implant and the surrounding cortical bone—these effects differed markedly, which was partially consistent with the conclusions of previous studies [40].

Table 3 summarizes the maximum equivalent elastic strain values in cortical bone across all models. The results indicated that in the RB thread group, the M3 implant exhibited the smallest maximum elastic strain value, while in the B thread group, the M5 implant showed the smallest maximum elastic strain value. Additionally, although the maximum von Mises stress values of the M7 and M9 implants were similar, the maximum elastic strain value in the cortical bone region corresponding to the M9 implant increased significantly by 49.2% compared to the M7 implant. It was noteworthy that only the M7 implant had a maximum elastic strain value (5.05 × 10^3^ με) below 6.7 × 10^3^ με, which may be attributed to the M7 implant having the largest bone–implant contact area among all models due to its neck microthreads. Increasing the bone–implant contact area not only enhances the initial stability of the implant but also helps distribute functional loads and reduce local stress concentration [41,42]. Among implants with the same thread form but different face angles, the contact area of the microthreads increased as the face angle increased. Currently, it is believed that the thread face angle primarily influences the stress distribution around the jawbone by adjusting the ratio of compressive stress to shear stress exerted on the jawbone [43]. Both tensile and shear stresses have adverse effects on bone tissue, with bone tissue having the lowest tolerance for shear stress [44]. Although von Mises stress is a comprehensive reflection of tensile, compressive, and shear stresses and cannot directly indicate the specific proportion of shear stress, according to Hooke’s law, the value of von Mises stress can, to some extent, reflect the magnitude of strain. Based on the Mechanostat Theory, which posits that bone cells can perceive mechanical strain in local tissues and initiate corresponding biological responses depending on the strain interval [45], we identified deficiencies in the face angle design of the M1 and M6 implants. The comprehensive analysis above indicated that for the NDI (2.9 mm) neck design, the V thread performs optimally when its face angle parameter was set at 30 degrees. In the RB thread group, the optimal face angle parameter was 60 degrees, while in the B thread group, this parameter was 45 degrees. Narendrakumar et al. [40] compared V thread implants with thread angles of 20 degrees, 30 degrees, 45 degrees, and 60 degrees, and found that the optimal thread face angle was 45 degrees. This conclusion differed from the findings of the present study, which may be due to differences in implant diameter between the two studies. However, there was currently no research confirming whether the effects of adjusting the thread face angle on large-diameter implants are equivalent to those on NDI.

Table 4 summarizes the maximum equivalent elastic strain values of all titanium–zirconium alloy (Ti-zr) implants under clinically relevant functional loads. We found that the maximum equivalent elastic strain values for all models were less than 0.0030 (mm/mm). This indicates that narrow-diameter Ti-zr implants used in the premolar region can achieve favorable biomechanical outcomes. A substantial body of long-term clinical studies (some spanning 5–10 years) has confirmed the high survival and success rates of Ti-Zr NDI, which perform comparably to standard-diameter implants even in the posterior region where masticatory forces are greater [46,47]. Figure 5 presents the von Mises stress distribution across all abutments. The analysis results indicated that: in both the B thread group and the RB thread group, the influence of the face angle parameter on the maximum von Mises stress of the abutment was not significant, whereas in the V thread group, different face angle parameters exhibited a marginal influence on the maximum von Mises stress of the abutment. It was worth noting that existing studies have primarily used FEA to examine the influence of variations in abutment taper angles or various abutment design schemes on the distribution of their maximum von Mises stress [20,48], while research on the influence of thread form and face angle parameters on abutment stress remains notably lacking.

We evaluated nine NDI models with different thread parameters. The findings of this study can provide clinicians with a reference for selecting implants with appropriate thread parameters in cases where the posterior region presents severe alveolar ridge width deficiency, patients are unsuitable for bone augmentation surgery, or when bone quality is poor (Type III bone). In this study, all results were derived from finite element analyses based on static structures. Although it has been found that different thread forms and their face angles can produce differential effects on the stress distribution in cortical bone, and that each thread configuration has an optimal thread face angle parameter that matches it, a universal mathematical model or empirical calculation formula capable of predicting the relationship between thread face angle and maximum von Mises stress has not yet been established. In the future, not only will multi-center validation studies be needed to enhance the reliability of the research findings, but verification must also be conducted through in vitro mechanical testing and animal experiments.

Novelty Statement:

1. First FEA-based optimization of thread form and face angle parameters for NDI (2.9 mm) in Type III bone (Figure 2).
2. Quantitative analysis of NDI’s thread form and face angle on abutment stress (Figure 6).
3. Identified NDI’s trapezoidal thread with 30 degrees face angle showing minimal microstrain in cortical bone (Table 3).

## 5. Conclusions

Although we strictly controlled the experimental conditions to ensure the reliability of the results as much as possible, this study still has certain limitations. For example, the jawbone was simplified as an isotropic material and the bone remodeling process of the jaw after implant placement was not considered. These aspects will be the focus of further research in the future. Based on the static finite element model established in this study, we draw the following conclusions:

1. For NDI (2.9 mm), the trapezoidal thread and face angle parameters (30 degrees) represented the optimal thread design for the neck region.

2. Each thread configuration had an optimal thread face angle parameter that matched it. In the RB thread group, the optimal thread face angle parameter was 60 degrees; in the B thread group, this optimal thread face angle parameter was 45 degrees.

3. In both the B and RB thread groups, the face angle parameter demonstrated negligible influence on the maximum von Mises stress of the abutment. In contrast, within the V thread group, variations in the face angle parameter exhibited a marginal yet measurable effect on the maximum von Mises stress of the abutment.

## Figures and Tables

**Table 1:** Mechanical properties and element size.

Material	Elastic Modulus [MPa]	Poisson Ratio	Element Size [mm]	References
Cortical bone	13,700	0.30	1.5	[23]
Cancellous bone (Type III)	1300	0.30	0.50	[24]
Ti-Zr implant	98,000	0.25	0.40–0.60	[25]
Abutment (Ti-6Al-4V)	110,000	0.35	0.50	[26]
IPS e.max ZirCAD framework	220,000	0.30	0.50	[27]

**Table 2:** The thread types, face angle parameters, number of elements, and number of nodes for each finite element model.

Model	Thread Form	Face Angle [degrees]	Elements	Nodes
M1	Reverse Buttress Threads	30	328,341	471,297
M2	Reverse Buttress Threads	45	325,273	471,099
M3	Reverse Buttress Threads	60	327,909	470,557
M4	Buttress Threads	30	328,423	471,426
M5	Buttress Threads	45	328,202	470,900
M6	Buttress Threads	60	328,287	471,093
M7	Trapezoidal Threads	30	330,032	473,857
M8	Trapezoidal Threads	45	325,293	466,305
M9	Rectangle Threads	90	328,101	470,647

**Table 3:** Maximum equivalent elastic strain in cortical bones of finite element analysis models.

Model	Vertical Load Condition	Oblique Load Condition
M1	2.04	7.18
M2	2.09	8.21
M3	2.11	7.07
M4	1.65	8.68
M5	1.98	7.46
M6	2.81	12.6
M7	2.21	5.05
M8	2.28	6.78
M9	1.53	9.93

Maximum equivalent elastic strain: ×10^3^ με.

**Table 4:** The maximum equivalent elastic strain of the implant in the finite element models.

Model	Vertical Load Condition	Oblique Load Condition
M1	5.26	22.39
M2	8.12	21.14
M3	7.62	23.82
M4	6.34	21.47
M5	5.88	19.71
M6	7.44	25.20
M7	7.47	23.24
M8	6.41	23.89
M9	6.00	22.35

Maximum equivalent elastic strain: ×10^−4^ mm/mm.

## Data Availability

The original contributions presented in the study are included in the article, further inquiries can be directed to the corresponding author.
